# Anthocyanins and Metabolic Disease: A New Frontier in Precision Nutrition

**DOI:** 10.3390/antiox15010061

**Published:** 2026-01-01

**Authors:** Giuseppe T. Patanè, Ruben J. Moreira, Maria de Almeida-Santos, Stefano Putaggio, Davide Barreca, Pedro F. Oliveira, Marco G. Alves

**Affiliations:** 1 Department of Chemical, Biological, Pharmaceutical and Environmental Sciences, University of Messina, 98166 Messina, Italy; giuseppe.patane@studenti.unime.it (G.T.P.); stefano.putaggio@studenti.unime.it (S.P.); davide.barreca@unime.it (D.B.); 2 Department of Medical Sciences, Institute of Biomedicine (iBiMED), University of Aveiro, 3810-193 Aveiro, Portugal; rubenjesusmoreira@ua.pt (R.J.M.); malmeidasantos@ua.pt (M.d.A.-S.); 3 LAQV-REQUIMTE, Department of Chemistry, University of Aveiro, 3810-193 Aveiro, Portugal; p.foliveira@ua.pt

**Keywords:** anthocyanins, flavonoids, obesity, diabetes mellitus, precision nutrition, inflammation, oxidative stress

## Abstract

Metabolic syndrome (MetS) represents a global health challenge mainly driven by chronic low-grade inflammation and persistent oxidative stress (OS). Current therapeutic and nutritional strategies often fail to resolve these interconnected core pathologies due to the multifactorial nature of MetS. Anthocyanins (ACNs), a class of potent dietary flavonoids, offer significant promise due to their established pleiotropic effects, including robust antioxidant activity through modulation of the Nrf2/ARE pathway, anti-inflammatory effects via NF-κB suppression, and overall support for glucose and lipid homeostasis. However, the therapeutic efficacy of ACNs is characterized by interindividual variability, which is intrinsically linked to their low systemic bioavailability. This heterogeneity in the response is due to the complex interplay between genetic polymorphisms affecting absorption, distribution, metabolism, and excretion (ADME), as well as the specific biotransformation capacity of the gut microbiome. This review proposes that achieving the full clinical potential of ACNs requires moving beyond conventional nutritional advice. We propose that precision nutrition, which integrates multi-omics data (e.g., genomics, metagenomics, and metabolomics), can determine the individual phenotype, predict functional metabolic response, and tailor safer and effective ACN-rich interventions. This integrated, multifactorial approach is essential for optimizing the antioxidant and metabolic benefits of ACNs for the prevention and management of MetS and its associated pathologies.

## 1. Introduction

Metabolic syndrome (MetS) is a cluster of interrelated metabolic pathologies that lead to biochemical and physiological imbalances. It was first defined in 1998 by the World Health Organization (WHO), which emphasized the strong implication of insulin resistance (IR) in its diagnosis. Based on this definition, the diagnosis of MetS requires the presence of insulin resistance (IR) along with the concurrent manifestation of at least two of the following conditions: hypertension, central obesity, hyperlipidemia, and microalbuminuria [1]. The coexistence of these metabolic alterations leads to increased risk of developing type 2 diabetes mellitus (T2DM), as well as atherosclerotic and cardiovascular diseases [2]. In recent years, its incidence has risen to epidemiological proportions, with approximately 25% of the global population affected. By 2030, MetS-associated cardiovascular diseases are projected to cause about 23.6 million deaths worldwide, underscoring the need for improved preventive and therapeutic strategies [3,4].

The development of MetS may arise from hereditary factors, as in rare genetic disorders such as Hurler syndrome or phenylketonuria, or be triggered through continuous exposure to environmental stressors or unhealthy habits [5,6]. Sedentary behavior, obesity, and unhealthy dietary patterns are key contributors to this growing prevalence [7,8]. Obesity, along with IR (observed in nearly 45% of individuals with obesity) promotes a chronic inflammatory state, which represents a major predisposing factor for the development of MetS and T2DM [9].

At a molecular level, strong evidence supports a close relationship between metabolic imbalance, the release of pro-inflammatory factors (Tumor Necrosis Factor Alpha (TNF-α)/Interleukin (IL)-6) and oxidative stress (OS), often in association with genetic alterations. Patients with MetS exhibit increased production of reactive oxygen and nitrogen species (ROS and RNS), due to a reduced catalytic activity of key antioxidant enzymes. This includes superoxide dismutase (SOD), endothelial nitric oxide synthase (eNOS), catalase (CAT), glutathione peroxidase (GPx), and glutathione-S-transferase [10,11]. During redox imbalance, the excessive RNS—peroxynitrite and nitrogen oxides—not only induce post-translational protein modifications but also disrupt physiological nitric oxide (NO) levels. This compromises vascular function, promoting cardiovascular complications in patients with MetS [12]. The comorbidities in MetS result in a strongly pro-oxidative environment, triggering structural damage to lipids and proteins due to oxidative damage, further aggravating cellular and metabolic dysfunction. Increased ROS and lipid peroxidation impair the antioxidant activity of high-density lipoproteins (HDLs) and enhance oxidation of low-density lipoproteins (LDLs), promoting the risk of atherosclerosis and myocardial infarction [13,14]. Moreover, protein peroxidation and carbonylation, together with glutathione (GSH) depletion, highlight the chronic OS state in MetS, supporting the use of oxidative damage biomarkers as diagnostic tools [15]. Under physiological conditions, ca. 2% of mitochondrial oxygen contributes to ROS generation, especially via complexes I and III of the electron transport chain. In individuals consuming carbohydrate- and energy-rich diets, ROS production is amplified, exacerbating cellular dysfunction [16,17]. Within mitochondria, excess triglycerides (TG) inhibit the adenosine nucleotide translocator, altering the Adenosine Triphosphate/Adenosine Diphosphate (ATP/ADP) ratio, disrupting oxidative phosphorylation, and enhancing superoxide (O^2•−^) production, perpetuating a chronic state of OS [18]. This oxidative imbalance drives the onset and progression of conditions such as T2DM, cardiovascular disease, central obesity, and IR. Autophagy dysfunction further exacerbates this metabolic imbalance across MetS components. Autophagy, the cellular process degrading damaged organelles and lipid droplets via lysosomes, is impaired in metabolic diseases. Defective autophagic fluxes particularly drive nonalcoholic fatty liver disease (NAFLD) progression through lipid accumulation, mitochondrial dysfunction, and ER stress, amplifying OS and inflammation [19]. Intermittent fasting-induced autophagy normalization confers hepatic protection, while selective autophagy emerges as a key therapeutic target in hepatic diseases. Notably, Nrf2 and AMPK pathways—both impaired in MetS—directly regulate autophagic flux, positioning autophagy as a central therapeutic target [20].

Current therapeutic strategies aim not only to control blood pressure and glycemia but also to reduce inflammatory and oxidative states, through both pharmacological approaches (e.g., statins, bile acid sequestrants, ezetimibe and resins) and lifestyle and nutritional modifications [21,22]. However, generalized dietary recommendations often fail to account for interindividual variability in metabolism, genetics, and microbiome composition, resulting in heterogeneous responses. This highlights the need for personalized nutrition capable of modulating specific molecular mechanisms [23]. In this context, anthocyanins (ACNs) have emerged as promising natural compounds. Their potential extends beyond antioxidant activity, positioning them as modulators of metabolic pathways and redox-related processes. This narrative review aims to elucidate the molecular mechanisms of ACNs in modulating OS, inflammation, and metabolic pathways in MetS; examine interindividual response variability driven by genetics, gut microbiota, and food matrix effects; and propose a precision nutrition framework integrating multi-omics data to reposition ACNs within metabolic medicine for personalized prevention and management.

## 2. Materials and Methods

This narrative review was conducted by systematically investigating the most relevant literature in the scientific databases PubMed (https://pubmed.ncbi.nlm.nih.gov, accessed on 1 August 2025), ScienceDirect (https://www.sciencedirect.com, accessed on 5 August 2025), and Google Scholar (https://scholar.google.com, accessed on 10 August 2025). Relevant articles published between 2010 and 2025 were considered, with particular emphasis on studies published in the last six years (2019–2025). The literature research combined multifactorial keywords reflecting three conceptual domains: (1) metabolic disease and associated pathologies, (2) anthocyanins and structural characteristics, and (3) mechanistic and physiological endpoints. Specific search strategies included the following combinations: “anthocyanins” AND (“metabolic syndrome” OR “obesity” OR “diabetes mellitus” OR “insulin resistance” OR “cardiovascular disease”), “anthocyanins” AND (“oxidative stress” OR “inflammation” OR “antioxidant”), “anthocyanins” AND (“bioavailability” OR “metabolism” OR “gut microbiota”), “anthocyanins” AND (“precision nutrition” OR “nutrigenomics” OR “personalized nutrition”), “flavonoids” AND (“metabolic disease” OR “MetS”), and “polyphenols” AND (“absorption” OR “ADME” OR “bioactivation”). Articles were evaluated based on the following inclusion criteria: (1) original experimental studies (in vitro or in vivo) demonstrating biochemical insights into anthocyanin action; (2) clinical trials and observational studies assessing physiological endpoints relevant to metabolic disease; (3) review articles and systematic reviews providing comprehensive synthesis of the literature. Exclusion criteria included studies with inadequate description of experimental design, methodology, or results; studies focusing exclusively on non-anthocyanin compounds without comparative data; and articles not directly addressing the role of anthocyanins in metabolic health or precision nutrition contexts. Priority was given to recent studies (2019–2025) addressing contemporary themes such as gut microbiota-mediated metabolism, nutrigenomics, metabolomics biomarkers, food matrix effects on bioavailability, and precision nutrition frameworks. However, seminal studies (2010–2018) establishing the fundamental mechanisms of anthocyanin action, genetic polymorphisms in absorption/metabolism, and clinical efficacy were also retained to provide a comprehensive mechanistic context.

## 3. The Dual Challenge of Metabolic Disease and One-Size-Fits-All Nutrition

To date, no specific guidelines exist for the treatment of MetS. Clinical recommendations are generally adapted from those for the individual conditions comprising the syndrome, such as T2DM, obesity, and cardiovascular disease. The main guidelines are issued by organizations including the American Association of Clinical Endocrinologists (AACE), the American Diabetes Association (ADA), the American Heart Association (AHA) and the National Heart, Lung, and Blood Institute (NHLBI), as well as several European bodies [24,25,26,27]. Although useful, these recommendations are often generic and focus on basic principles such as reducing total energy intake, limiting saturated and trans fats, and promoting regular physical activity [28]. The common goal is to decrease physical parameters such as waist circumference, abdominal diameter, and fat mass, improve glycemic control, and reduce cardiovascular risk factors. A recent systematic review of 2684 articles reported that in 73% of the patients, a multidisciplinary approach involving dietary modifications and physical activity led to regression of obesity and metabolic disorders [29]. Nonetheless, standardized dietary approaches are not universally effective. For instance, the Dietary Guidelines for Americans (DGA, 2015–2020) recommended limiting saturated fatty acids to <10% of daily energy intake to reduce cardiovascular risk and LDL-cholesterol [30,31]. Yet, a clinical study on more than 1000 Iranian women reported that those not adhering to DGA recommendations had a 28% lower risk of developing metabolic disorders compared with those who followed the guidelines more strictly [32]. The Mediterranean Diet, despite its cardiovascular and metabolic benefits, does not consistently prevent the onset of MetS. Moreover, higher intakes of oleic and α-linolenic acids, despite their beneficial effects on cardiovascular health and glucose metabolism, have been linked to an increased incidence of MetS [33,34,35,36]. This divergence is also valid for the Dietary Approach to Stop Hypertension (DASH), which has yielded variable efficacy in managing metabolic parameters such as TG, HDL-cholesterol, and glycemia [37,38,39]. These inconsistencies highlight the limitations of “one-size-fits-all” dietary approaches. Interindividual variability, stemming from genetic, behavioral, and metabolic differences, plays a crucial role in response to diet. Integrating omics can help tailor dietary interventions, monitor adherence, and identify biomarkers reflecting metabolic changes [40,41,42]. It is also worth noting that dietary biomarkers may reflect how specific foods influence physiology and disease management, since these not only exert beneficial metabolic effects but also modulate their own absorption and consequent metabolism playing a role in precision nutrition [43,44]. Among bioactive compounds, ACNs that can be found in colorful fruits and vegetables are promising compounds, due to their antioxidant, anti-inflammatory, and anti-hypertensive activities [45,46,47]. MetS is consistently associated with elevated OS biomarkers, including 8-hydroxy-2-deoxy-guanosine, 8-epiprostaglandin F2α, adipokines, and advanced glycation end products (AGEs), highlighting the potential of ACNs to mitigate disease progression [15,48,49]. Clinical and epidemiological studies show that ACN-rich foods or supplements can reduce central obesity, modify the lipid profile, and improve postprandial glycemic control in T2DM patients [50,51,52,53,54]. The following sections explore the absorption and metabolism of this subclass of polyphenols, their effects on metabolic pathways and OS, and their potential role in precision nutrition strategies for MetS management.

## 4. Anthocyanins: From Dietary Pigments to Bioactive Compounds

Before exploring how ACNs modulate the pathophysiology of MetS, it is essential to outline their natural sources, chemical structure, and bioavailability. ACNs, a subclass of flavonoids, are the largest group of water-soluble pigments, which are responsible for the vibrant colors of many fruits, vegetables, and grains, as well as for their astringent taste [55,56]. They are particularly abundant in berries, such as blueberries (3.87–4.87 mg/g), black goji berries (6.25 mg/g), and grapes (0.40–0.70 mg/g) [57,58]. Chemically, ACNs consist of an anthocyanidin aglycone (two aromatic rings (A, B) and a heterocyclic ring (C), forming a C6-C3-C6 backbone-2-phenylbenzopyrylium) linked to, for instance, the following sugars: glucose, galactose, arabinose, xylose, and rhamnose [46,59]. More than 550 aglycone forms have been identified to date, with cyanidin, peonidin, malvidin, delphinidin, and petunidin being the most common [60]. The degree, type, and position of glycosylation, as well as conjugation with aliphatic and aromatic acids, determine the type of ACN. Estimated average dietary intake varies geographically as follows: Europe (15–65 mg/day), China (50 mg/day), Finland (~150 mg/day), and the United States (20–215 mg/day). However, in the US, this often reflects red wine consumption rather than a diet rich in fruits and vegetables [61,62,63]. ACNs have low bioavailability (2–12%), depending on the aglycone type, sugar moieties, and molecular weight, and their effects are largely mediated by their metabolites rather than the starting compounds [63,64,65]. In vivo studies show that less than 0.1% of ingested ACNs are absorbed unchanged, whereas phenolic metabolites reach markedly higher plasma levels [63,66].

**Figure 1:**
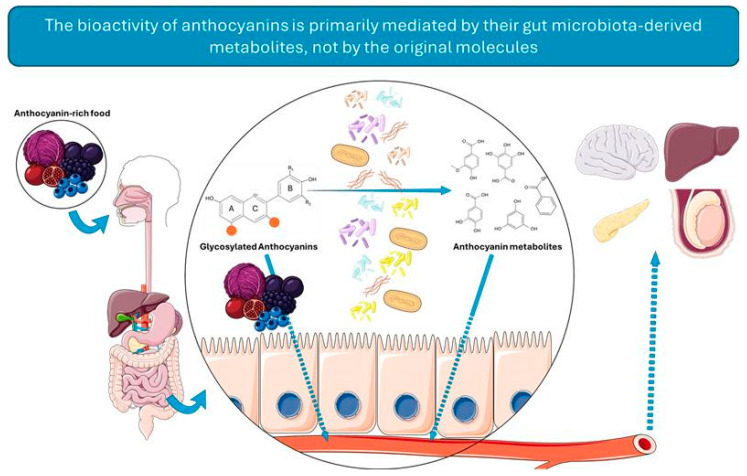
Intestinal biotransformation and absorption of anthocyanins. This figure illustrates the metabolic fate of dietary anthocyanins following ingestion. While glycosylated anthocyanins exhibit low direct bioavailability, they pass to the colon where they interact with the gut microbiota. Commensal bacteria enzymatically degrade anthocyanins, converting them into smaller, bioavailable phenolic acid metabolites (e.g., protocatechuic acid, vanillic acid). Unlike the parent molecules, these metabolites are readily absorbed across the intestinal epithelium, entering the systemic circulation to reach target organs such as the brain, liver, pancreas, and reproductive tissues, where they mediate biological effects. Adapted from Servier Medical Art (https://smart.servier.com), licensed under CC BY 4.0 (https://creativecommons.org/licenses/by/4.0/).

The journey of an ACN from ingestion to systemic circulation is mediated by a series of transport proteins. Absorption begins in the stomach, where mono- and di-glucosyl ACNs can be rapidly absorbed via the organic anion carrier bilitranslocase [67,68]. In the small intestine, key transporters, such as sodium–glucose cotransporter 1 (SGLT1), glucose transporter (GLUT) 2, and organic anion transporting polypeptide 2B1 (OATP2B1) mediate uptake into enterocytes [69,70,71]. SGLT1 enables active transport for the uptake of intact ACN glycosides, which may subsequently be hydrolyzed intracellularly [72]. GLUT 2 facilitates the bidirectional transport of both aglycones and glycosylated forms across the basolateral membrane. OATP2B1, a broadly expressed transporter, contributes to the absorption of all forms of ACNs along with various drugs and endogenous compounds [73]. The deglycosylation by intestinal β-glucosidases further regulates absorption [72]. These include lactase-phlorizin hydrolase (LPH), encoded by *LCT*, located at the brush border membrane of small intestine, and cytosolic β-glucosidase, encoded by *GBA3*, which acts within the enterocytes after SGLT1-mediated uptake [74]. As will be explored later in this review, the composition of gut microbiota also influences metabolism, since several bacteria also possess enzymes capable of hydrolyzing and transforming these compounds [75]. Figure 1 summarizes the intestinal biotransformation and absorption of ACNs, a critical step for ACN bioactivity.

Most data on ACN absorption and transport are derived from rat studies or human-relevant cell models, with limited clinical data available [76,77]. A major determinant of their low systemic bioavailability is microbial metabolism in the gut. Bacterial enzymes not only deglycosylate ACNs but also cleave the A and B benzoic rings, producing phloroglucinol and benzoic derivatives [78]. Using human microbiota-associated and germ-free rats, it was demonstrated that cyanidin 3-O-glucoside is completely metabolized in the presence of bacterial enzymes, primarily into 2,4,6-trihydroxybenzaldehyde and 2,4,6-trihydroxybenzoic acid, while remaining largely unchanged in the absence of these enzymes. Similarly, direct incubation of ACNs with *Clostridium saccbarogumia* and *Eubacterium ramulus* led to over 80% degradation after 48 h, driven by α-galactosidase, α-rhamnosidase, and β-glucosidase activity [79]. Beyond deglycosylation, gut microbes also catalyze deacetylation and ring-cleavage reactions, yielding phenolic acids, such as protocatechuic acid (PCA) from cyanidin, syringic acid from malvidin, vanillic acid from peonidin, and 4-hydroxybenzoic acid from pelargonidin [80]. Additional metabolites, including resorcinol, catechol, and caffeic derivatives, have been identified using ^13^C-labeled ACNs [63,69,81]. Despite extensive research, no study has yet fully mapped the stepwise metabolic fate of ACNs.

Following intestinal metabolism and absorption, ACNs undergo phase II biotransformation, mainly glucuronidation, methylation, and sulfation, catalyzed by UDP-glycosyltransferases (UGTs), catechol-O-methyltransferase (COMT), and sulfotransferases (SULTs), which occur primarily in the liver and kidneys [70,71,82]. These reactions generally enhance the stability and water solubility of ACNs, dictating their distribution and excretion [83]. Although native ACNs exhibit low bioavailability, their metabolites are more bioaccessible and bioactive, contributing to the several beneficial health effects attributed to them.

## 5. Anthocyanins as Signaling Hubs in Metabolic Health

ACNs modulate metabolic health by regulating oxidative balance, gene expression, and signaling pathways such as insulin sensitivity (IS), inflammation, and lipid metabolism [84].

### 5.1. Orchestrating Inflammation and Oxidative Stress

Among the main mechanisms by which ACNs influence metabolic health is the modulation of inflammation and OS. A central player in these is the nuclear factor-kB (NF-κB) pathway, which regulates both cytokine production and DNA transcription [85]. NF-κB is a cytoplasmic complex composed of five subunits-NF-κB1 (p50), NF-κB2 (p52), RelA (p65), RelB, and c-Rel that remains inactive through binding to inhibitory IκB proteins, primarily IκBα. Upon phosphorylation by IκB kinases (IKKs), IκBα is degraded at the N-terminal serine residues, releasing NF-κB to translocate to the nucleus and activate the transcription of pro-inflammatory genes [86,87]. The free heterodimers, such as p50/RelA and p50/c-Rel, drive the production of pro-inflammatory cytokines (e.g., TNF-α,IL-1α, IL-1β and IL-10); chemokines such as IL-8, adhesion molecules (vascular cell adhesion molecule (VCAM-1), and intercellular cell adhesion molecule (ICAM-1)); and inducible nitric oxide synthase (iNOS), cyclooxygenase-2 (COX-2), and cytosolic phospholipase 2 [88,89].

ACNs have been shown to inhibit NF-κB activation and downstream inflammatory cascade. Pretreatment of 3T3-L1 murine adipocytes with cyanidin-3-O-glucoside (C3G) reduced nuclear NF-κB levels and IKK activity after exposure to palmitic acid [90]. Similarly, C3G, peonidin-3-O-glucoside, and PCA from purple rice exhibit potent anti-inflammatory effects by reducing IκBα degradation, lowering p65 levels, and blocking the kinase/mitogen-activated protein kinase (ERK/MAPK) signaling pathway [91]. In macrophages, ACNs such as p-coumaroyl and pelargonidin-3-O-glucoside prevent the translocation of p65 to the nucleus [92,93], while the dietary intake of ACNs from blueberries decreased serum levels of TNF-α, interferon gamma (IFN-γ), and p65 and increased IL-10 and IL-22 [94,95]. Inhibition of NF-κB also reduces COX activity, prostaglandin E2 (PGE2) production, and ROS-driven inflammation. Cyanidin-3-O-(2″-xylosyl)-glucoside suppresses activator protein 1 (AP-1) activation and regulates NO production and SOD activity [96,97,98]. Notably, the aglycone cyanidin activates nuclear factor erythroid 2-related factor 2 (Nrf2), a key regulator of OS and suppressor of apoptosis, via modulation of the NF-κB/MAPK pathway [99]. By inhibiting NOS, ACNs limit NO overproduction and peroxynitrite anion formation, reducing the pro-inflammatory state and improving IS [100,101,102,103].

Although the precise molecular mechanisms are not fully elucidated, macrophages and T cells play key roles in IR, by releasing type 1 cytokines (TNF-α, IL-1β and IFN-γ). These cytokines activate the IKK/NF-κB pathway, leading to serine phosphorylation of insulin receptor substrate 1 (IRS-1), which blocks the downstream insulin signaling transduction pathway [104,105,106,107,108,109]. By modulating NF-κB and MAPK pathways, ACNs reduce the release of TNF-α and/or IL-1β, restoring IS and counteracting metabolic dysfunction [84].

Beyond their anti-inflammatory role, ACNs exert promising antioxidant properties. Structurally, the presence of hydroxyl or methoxy groups on the flavylium core, particularly in catecholic (1,2-diphenol) or pyrogallolic (1,2,3-triphenol) B-ring configurations, enhances their capacity to scavenge ROS. Interaction with free radicals generates stable semiquinone intermediates, in which radical delocalization over the B and C rings neutralizes reactive species and disperses the charge [110]. This neutralization occurs via hydrogen atom transfer (HAT) and single-electron transfer (SET) mechanisms, in which a hydrogen atom or an electron is donated to stabilize the radical, respectively [111]. ACNs such as aurantinidin, capensinidin, cyanidin, and delphinidin are potent scavengers of O^2•−^ and hydroperoxyl radicals. Experimental evidence demonstrated that ACNs exhibit lower EC50 for DPPH and NO scavenging than ascorbic acid, highlighting their superior radical-neutralizing capacity relative to conventional antioxidants [112,113]. These chemical and functional properties underline the ability of these compounds to mitigate OS and protect cells from ROS-induced damage.

In addition to the already discussed antioxidant activity exerted by this class of molecules, cyanidin-3-galactoside can neutralize hydrogen peroxide formation through chelation of metal ions involving the C3- or C5-hydroxyl substituents or the hydroxyl groups in the ortho position in the B-ring [114]. They also enhance the abundance and activity of endogenous antioxidant enzymes, including CAT, GPx, and SOD, thereby protecting cells from OS [101,115,116,117]. These effects are linked to activation of the Nrf2 signaling pathway. Under OS, Nrf2 dissociates from its inhibitory protein Keap1 and translocates to the nucleus, forming a heterodimer with the Maf protein, and binding to the antioxidant response element (ARE), promoting the transcription of genes encoding antioxidant enzymes [118,119].

### 5.2. Regulating Glucose and Lipid Metabolism

The regulation of energy balance and metabolic processes relies on AMP-activated protein kinase (AMPK), a heterotrimeric complex composed of a catalytic α subunit and two regulatory subunits, β and γ [120]. Under stress conditions, an increased AMP/ATP ratio activates AMPK through binding of AMP to the γ subunit, thereby stimulating energy production and suppressing anabolic pathways [121,122]. Once activated, AMPK inhibits gluconeogenesis by downregulating glucose-6-phosphatase and phosphoenolpyruvate carboxykinase, while enhancing glycolysis and glycogenolysis via phosphofructokinase-2 activation and glycogen synthase inhibition, respectively [123,124,125]. In parallel, it also promotes fatty acid oxidation through the activation of carnitine acyltransferase I and limits lipid and cholesterol synthesis by inhibiting acetyl-CoA carboxylase, indirectly reducing fatty acid synthase and 3-hydroxy-3-metilglutaril-CoA reductase activity [126,127,128,129,130]. AMPK also increases glucose uptake by phosphorylating protein kinase B (PKB or Akt) on Ser789, which, in turn, phosphorylates AS160, leading to GLUT4 translocation to the plasma membrane [131,132,133,134]. In men with obesity, AMPK function is impaired, leading to reduced fatty acid oxidation and increased lipogenesis, limiting exposure of GLUT4, compromising glucose homeostasis, and promoting metabolic dysfunction [135,136].

ACNs counteract these effects by modulating phosphoinositide 3-kinase (PI3K)/Akt and AMPK pathways, restoring glucose and lipid homeostasis. In vitro, ACNs from black rice extracts promote GLUT4 translocation via the PI3K/Akt and AMPK/p38 MAPK pathways in C2C12 myoblast cells, enhancing the uptake of glucose [137]. Similarly, C3G from black soybeans and cyanidin-3-rutinoside increased glucose uptake in L6 myoblast cells and 3T3-L1 adipocytes, respectively. This effect is mediated by the activation of insulin signaling, including phosphorylation of IRS-1, activation of PI3K, and phosphorylation of Akt, which promotes GLUT4 expression and translocation [138,139]. These are particularly relevant in adipose tissue, where fat accumulation promotes OS and inflammation, impairing the PI3K/Akt pathway. ACNs counteract the effects by reducing ROS, inhibiting NF-κB, thus promoting the expression of endogenous antioxidant defenses, restoring IS and glucose uptake [135,140].

In mice fed a high-fat diet (HFD), ACNs from purple corn (400 mg/kg) reduced body weight gain, fat mass, cholesterol, and triglyceride. The beneficial effects are linked to their ability to activate AMPK and modulate gene expression, suppressing peroxisome proliferator-activated receptor (PPAR) γ, CCAAT/enhancer-binding protein α (C/EBPα), and sterol regulatory element-binding protein-1c (SREBP-1c), while upregulating PPARα, peroxisome proliferator-activated receptor gamma coactivator 1-alpha (PGC1α), PR domain-containing 16 (PRDM16), and fibroblast growth factor 21 (FGF21), inhibiting fat accumulation and promoting the breakdown of lipids [141,142]. ACNS (200 mg/kg) from purple potatoes similarly decreased hepatic triglyceride content by activating AMPK (promoting fatty acid oxidation) and inhibiting SREBP-1c (limiting de novo fatty acid synthesis) [143]. In HepG2 cells, C3G activates the AMPK in a concentration-dependent manner, leading to phosphorylation and inactivation of acetyl-CoA carboxylase and reduced malonyl-CoA levels. This process stimulates the expression of carnitine palmitoyl-CoA transferase 1, thus enhancing fatty acid oxidation in cells. These effects are avoided when AMPK is pharmacologically or genetically inhibited, confirming its central role [144]. Finally, since AMPK activity correlates with the AMP/ATP ratio, ACN-mediated AMPK activation may also improve mitochondrial function. Mice with AMPK mutations are physically less active, potentially due to a reduced content of mitochondria in muscle, suggesting that ACN action could support mitochondrial health and energy metabolism [145,146].

### 5.3. Modulating Cellular Communication

Endothelial dysfunction occurs when the endothelium is no longer able to maintain vascular homeostasis, leading to abnormal production of vasodilators, pro-inflammatory molecules, and thrombotic factors. This alteration contributes to thrombosis, inflammation, and cardiovascular diseases, such as atherosclerosis and hypertension [147], and represents an early marker of vascular pathology in atherosclerosis, diabetes, obesity, and more generally of MetS [148]. Under physiological conditions, endothelial cells produce and release vasodilators (e.g., NO, prostacyclin, endothelin) and respond to insulin. Insulin activates eNOS via the PI3K/Akt pathway, increasing NO production and promoting GLUT4 translocation. Endothelial dysfunction disrupts this transduction mechanism, contributing to IR [149]. LDLs further compromise endothelial function. Accumulation of ROS can lead to oxidation of LDLs, triggering the release of IL-1 and TNF-α, promoting the development of atherosclerosis [150]. LDL accumulation can also inhibit the transcription of NOS and the reduction in L-arginine uptake, inhibiting the synthesis of NO [151].

ACNs exert protective effects against cardiovascular diseases, specifically against endothelial dysfunction. In vivo evidence from apolipoprotein (APO) E-deficient (APOE-/-) mice showed that PCA, a major metabolite from ACN metabolism, alleviates endothelial dysfunction by reducing the VCAM-1 and ICAM-1, reducing monocyte adhesion and attenuating the development of atherosclerotic lesion [152]. In vitro, treatment of human endothelial cells with a mixture of ACNs and their gut metabolites inhibited TNF-α-related monocyte adhesion and MCP-1-mediated transmigration. These effects were associated with the modulation of genes regulating endothelial integrity, permeability, and cell adhesion, including *CLDN1*, *CXCL12* or *IKBKB*, *CDH5*, *ITGA5*, and *CAPN1* [153]. ACNs improve vascular tone/relaxation by increasing eNOS expression while suppressing endothelin-1 and endothelin-converting enzyme-1, improving vascular relaxation [154]. Additionally, the beneficial effects of ACNs on endothelial function may extend to their influence on gut microbiota and the regulation of trimethylamine N-oxide (TMAO) metabolism. TMAO is derived from the hepatic oxidation of trimethylamine (TMA) by flavin-containing monooxygenases [155]. It can impair endothelial function both through the inhibition of eNOS, the reduction in NO, and through the promotion of inflammation and cholesterol dysregulation [156]. Dietary supplementation with blackberry ACNs in HFD-fed mice reduced TMA and serum TMAO concentrations, along with the expression of NF-κB, thus alleviating lipid dysregulation and vascular inflammation [157].

In addition, an in vivo study conducted by Marques and colleagues demonstrated that blackberry extract, rich in ACNs, can regulate neuroinflammation. Specifically, the extract improves the composition of gut microbiota, counteracts HFD-induced dysbiosis, modulates tryptophan metabolism by increasing the production of kynurenic acid, and mitigates obesity-related neurological complications [158].

**Figure 2:**
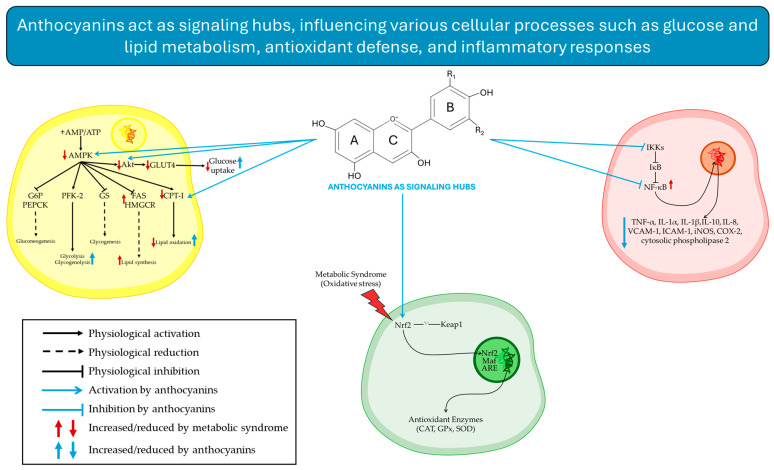
Modulation of metabolic health by anthocyanins action as signaling hubs. Anthocyanins regulate cellular pathways at different levels, including glucose and lipid metabolism (yellow cell), antioxidant defense (green cell), and inflammatory responses (red cell). These mechanisms (blue arrows) avoid the deleterious effects of metabolic syndrome (red vertical arrows). Regarding glucose and lipid metabolism, anthocyanins have a direct effect on AMPK (correlated with the increase in the AMP/ATP ratio), prompting glucose uptake, glycolysis, glycogenolysis, and lipid oxidation (blue vertical arrows). These also inhibit gluconeogenesis, glycogenesis, and lipid synthesis (blue vertical arrows). Abbreviation: ACC: acetyl-CoA carboxylase, Akt: protein kinase B, AMPK: AMP-activated protein kinase, ARE: antioxidant response element, CAT: catalase, COX-2: cyclooxygenase-2, CPT-1: carnitine palmitoyltransferase-1, FAS: fatty acid synthase, G6P: glucose-6-phosphatase, GLUT4: glucose transporter type 4, GPx: glutathione peroxidase, GS: glycogen synthase, HMGCR: 3-hydroxy-3-methylglutaryl-CoA reductase HO-1: geme oxygenase-1, ICAM-1: intercellular cell adhesion molecule, IκB: inhibitor of nuclear factor kappa-B, IKK: IκB kinase, IL: Interleukin, iNOS: inducible nitric oxide synthase, Keap1: Kelch-like ECH-associated protein 1, NF-κB: nuclear factor kappa-light-chain-enhancer of activated B cells, Nrf2: nuclear factor erythroid 2-related factor 2, PEPCK: phosphoenolpyruvate carboxykinase, PFK-2: phosphofrutokinase-2, ROS: reactive oxygen species, SOD: superoxide dismutase, TNF-α: Tumor Necrosis Factor-alpha, VCAM-1: vascular cell adhesion molecule. Adapted from Servier Medical Art (https://smart.servier.com), licensed under CC BY 4.0 (https://creativecommons.org/licenses/by/4.0/).

Overall, ACNs modulate multiple key signaling hubs and are involved in a variety of cellular signaling pathways. They exert potent antioxidant and anti-inflammatory effects through both direct and indirect mechanisms, particularly those involving HAT, SET, and the Nrf2/ARE pathway. ACNs also enhance IS, glucose uptake, and lipid metabolism by regulating the PI3K/Akt and AMPK/p38 MAPK pathways, while also supporting mitochondrial health and improving vascular tone and relaxation. Collectively, these actions underscore the versatility of ACNs in influencing diverse physiological processes and highlight their potential applications in managing metabolic disorders and their use in precise nutrition settings (Figure 2).

## 6. The Framework for Precision Nutrition: A Multifactorial Perspective on Individual Responses to Anthocyanins

The concept of precision nutrition marks a paradigm shift from generalized dietary guidelines towards tailored nutritional strategies designed to optimize health and mitigate disease risk at an individual level. Although often used interchangeably with personalized nutrition or nutrigenomics, precision nutrition is a more holistic and comprehensive approach, whereas personalized nutrition primarily focuses on incorporating genomic data to complement clinical history and biometric assessment. Thus, precision nutrition encompasses a multidimensional framework integrating two broad categories of data: (1) individual-level data (phenotypic and biometric information, clinical history, lifestyle factors such as dietary habits, eating patterns, circadian rhythms, and physical activity, and psychosocial and socioeconomic characteristics) and (2) biological and molecular data (genomics, metagenomics, and metabolomics) [159]. This integrative approach is essential for understanding why individuals exhibit considerable variability in their response to dietary interventions, particularly those involving bioactive compounds such as ACNs. The efficacy of ACNs in modulating metabolic health is not uniform; rather, it reflects the predictable outcome of a complex interplay among genetic background, gut microbiome, the delivery and bioavailability of ACNs, unique metabolic signatures, and overarching lifestyle factors [83]. This section aims to dissect these interdependent pillars, providing a comprehensive framework to understand the multifactorial nature of interindividual responses to ACNs.

### 6.1. Genetic Polymorphisms in Anthocyanin Absorption and Pre-Systemic Metabolism

Phenotypic data, such as body weight, glycemia, and other biometric indicators, provide an essential foundation for tailoring dietary plans to improve metabolic health. However, phenotype alone does not show the full complexity regarding the nutritional health of a patient, nor does it indicate how they will respond to a specific nutritional plan, such as the inclusion of an ACN-rich diet. Beyond these observable traits, genetics provides the foundational blueprint that dictates an individual’s inherent capacity to absorb, metabolize, and distribute dietary ACNs. This genetic layer establishes a baseline predisposition that can either facilitate or impede the bioactivity of these compounds, long before other factors come into play.

While nutritional interventions based solely on current diet, phenotype, or genotype can yield short-term improvements in eating habits over six months, the inclusion of genomic information, such as risk single-nucleotide polymorphisms (SNPs) for MetS, obesity, and diabetes, may offer additional unforeseen benefits [138]. For instance, incorporating genetic information regarding *APOE* and *TCF7L2* into personalized nutritional programs promotes the exclusion of discretionary foods and beverages contributing to an improved dietary quality [139]. However, the predictive power of genetic information depends strongly on the selected gene panel and population context. An example of this is a study that only included the *FTO* gene for personalized diet advice, which did not change healthy eating motivation [140]. These genes have polymorphisms associated with MetS risk factors. For instance, variants in *FTO*—such as rs9939609 and rs1421085—are linked to increased consumption of sugar and saturated fat [160] and the latter to loss-of-control eating behaviors [161]. Similarly, *TCF7L2* rs7903146 not only increases T2DM risk [162] but also influences total energy intake [163] and the success of lifestyle-induced weight loss [164]. *APOE* variants (rs429358 and rs7412) independently increase risk for T2DM and obesity [165], whereas *MC4R* may promote susceptibility to obesity through appetite dysregulation [166]. There are additional polymorphisms that further illustrate the complex genetic landscape influencing metabolic risk: *PPARG* rs1801282, linked to higher body mass index (BMI) and hypercholesterolemia [167]; *PPARA* rs1800206, correlated with elevated C-reactive protein (CRP) in individuals with overweight/obesity [168]; *PLIN1* rs894160, affecting cell lipid metabolism and increasing the waist-to-hip ratio [169]; *GCKR* rs1260326, linked to impaired hepatic lipid and glucose metabolism, leading to NAFLD and IR [170]; and lipoprotein lipase (*LPL*) rs328, modulating adipokine and endocrine profiles [171]. Collectively, the presence of multiple risk variants correlates with greater susceptibility to MetS, higher BMI, and increased central adiposity [172]. This pre-existing genetic susceptibility creates the physiological context into which ACNs are introduced, and their efficacy may be influenced by the very pathways these polymorphisms affect.

#### 6.1.1. Pharmacogenetics of Anthocyanin Absorption, Distribution, Metabolism, and Excretion

Beyond general metabolic risk, genetic variations in the genes encoding transporters and enzymes involved in the absorption, distribution, metabolism, and excretion (ADME) of ACNs are critical determinants of their bioavailability. These polymorphisms can create distinct subgroups within the population with vastly different abilities to process these bioactive compounds. Although current research regarding ADME of ACNs is sparse, it is possible to infer potential effects by knowing which genetic variations are present in the patient’s genetic makeup, thus predicting their response.

Absorption of ACNs can begin in the stomach, where bilitranslocase is thought to mediate uptake [67,68]. However, the gene encoding this transporter has not yet been definitively identified, representing a bottleneck in assessing its genetic variability and its relevance in precision nutrition.

In the small intestine, SGLT1, GLUT2, and OATP2B1, respectively, encoded by the *SLC5A1* [173], *SLC2A2* [174], and *SLCO2B1* [175] genes, are involved in intestinal absorption of ACNs. While direct studies on how *SLC5A1* polymorphisms affect the transport of ACNs are limited, insights can be drawn from research on its primary substrate, glucose. Several *SLC5A1* missense mutations, including rs17683011, rs17683430, and rs33954001, reduce transporter capacity, while rs933026071, rs765502638, rs779428134, rs747215838, and rs1304151494 result in complete loss of activity [176,177]. Given that *SGLT1* also mediates uptake of flavonoid glycosides through the mechanism analogous to glucose transport [178], it is highly probable that these functional variants similarly impair the intestinal uptake of glycosylated ACNs. This provides a direct, testable hypothesis for precision nutrition: individuals carrying these loss-of-function variants in *SLC5A1* may be “poor absorbers”, exhibiting lower systemic levels of the parent compounds and potentially altered metabolite profiles produced by colonic metabolites from the unabsorbed flavonoids; identifying those variants could thus be valuable for optimizing interventions.

The genetic variation in *SLC2A2* illustrates the wide spectrum of genetic effects, from severe, rare mutations to common polymorphisms with subtle metabolic impacts. A total of 70 different *SLC2A2* variants, including missense, nonsense, and frameshift mutations, are known to cause Fanconi–Bickel syndrome. This is a severe and rare autosomal recessive disorder of carbohydrate metabolism characterized by a non-functional or absent GLUT2 protein [179]. In such individuals, the absorption of ACNs reliant on this transporter would be abolished, representing a clear case of genotype-driven non-response. Conversely, common SNPs, such as rs5393, are associated with an increased risk of progression from impaired glucose tolerance to T2DM [180], highlighting how *SLC2A2* variation can influence nutrient handling and disease susceptibility. This underscores the necessity of variant-specific interpretation within a precision nutrition framework.

Functional characterization of common missense variants in *SLCO2B1* has identified several variants with altered transport activity. An in vitro study demonstrated that the rare variant rs1009122956 overturned OATP2B1-mediated uptake of all tested substrates almost completely, without changing protein abundance. Meanwhile, the more common variants rs12422149 and rs2306168 significantly reduce transport of various substrates, including estrone sulfate and rosuvastatin [175]. These loss-of-function variants likely diminish the intestinal absorption of ACNs, defining a subgroup of “low OATP2B1-mediated uptake” individuals. However, the relationship between genotype and phenotype of *SLCO2B1* is complex. For instance, rs12422149, despite showing wild-type (WT) activity in vitro, has been associated with higher plasma concentrations of endogenous OATP2B1 substrates in vivo [181]. This discrepancy illustrates the multifactorial nature of nutrient–genotype interactions and emphasizes the need for integrated, multi-level data rather than reliance on single-gene effects alone.

#### 6.1.2. Interindividual Variability in Intestinal Deglycosylation Enzymes

Genetic variations affecting intestinal β-glucosidases can impact the overall bioavailability and the metabolic fate of these dietary compounds. While the primary role of LPH is the digestion of lactose [182], it also possesses phlorizin hydrolase activity, enabling it to hydrolyze a range of dietary flavonoid glycosides [183]. This activity, in the active site of this enzyme, makes LPH a critical gatekeeper for the absorption of flavonoid aglycones [184]. The regulation of the *LCT* gene expression represents one of the most well-characterized examples of gene–diet interaction. The persistence or non-persistence of lactase expression is not caused by mutations in *LCT* itself, but by cis-regulatory SNPs located in an enhancer region of the adjacent *MCM6* gene [185]. The key SNP, rs4988235, determines this trait: individuals with the ancestral CC genotype exhibit post-weaning downregulation of *LCT* transcription and very low LPH levels (lactase non-persistence-lactose intolerance), whereas carriers of the T allele maintain high enzyme expression adulthood [186,187,188]. Given that the majority of adults worldwide are lactase non-persistent [189], low LPH activity may also translate into a reduced capacity to deglycosylate ACN glycosides at the brush border, likely resulting in lower absorption of their aglycones and attenuated systemic bioavailability from this primary pathway.

A remarkable feature of the *GBA3* gene in humans is that it is a polymorphic pseudogene [190]. A common SNP, rs358231 (T>A), introduces a premature stop codon, producing a catalytically inactive truncated protein, with the terminal α-helix necessary for proper folding [190]. This loss-of-function variant represents a binary mechanism for interindividual variability: homozygotes for the A allele lack a functional cytosolic β-glucosidase enzyme [191]. Consequently, the deglycosylation of ACNs would rely solely on the activity of LPH and the metabolic capacity of the gut microbiota. The frequency of this null allele varies significantly across populations, being more frequent in Eurasia, possibly reflecting evolutionary pressures related to historical dietary patterns [190]. This inactivating polymorphism provides a strong genetic basis for ethnic differences in flavonoid metabolism and underscores the need for population-specific precision nutrition strategies. A combined analysis of genotypes for *MCM6* (regulating LPH) and *GBA3* could identify individuals who are “poor metabolizers” of dietary flavonoid glycosides, as both of their major intestinal deglycosylation pathways would be compromised.

### 6.2. Genetic Polymorphisms in Post-Absorption Phase II Metabolism

In the context of precision nutrition, SNPs affecting UDP-glucuronosyltransferases, COMTs, and SULTs have not yet been integrated. However, several polymorphisms that reduce the expression or activity of these key proteins could be relevant when predicting ACN metabolism and bioavailability.

For UGTs, SNPs that reduce enzymatic activity (e.g., rs4148323, rs17868323, rs17863778, rs17868324, rs11692021, rs1042597, rs17863762, rs13119049, and rs12233719) or reduce its expression (rs3064744, rs4124874, and rs10929302) exhibit population-specific prevalence among Africans, Asians, and Caucasians [192]. The best-characterized COMT polymorphism, rs4680, although not solely responsible for COMT’s phenotypical variance, decreases enzyme activity by 35 to 40% when compared to the WT allozyme, due to higher thermolability [193]. Regarding sulfation of flavonoids, SULTA1 mostly metabolizes epicatechin, while SULT1C4 acts on quercitrin; both metabolize rutin [194]. The SULT1A1 variant rs544820732 decreases activity by 13%, whereas other SNPs (rs9282861, rs544820732, rs767487725, rs9282861, rs758145522, rs28374453) lower activity by at least 49% [194]. Similarly, rs769869249 and rs749518195 in SULT1C4 decrease activity by at least 30% [194].

Collectively, these SNPs represent key determinants of interindividual variability in ACN metabolism, ultimately leading to lower bioavailability. When considering ACN administration as a potential therapy for MetS-adjacent pathologies, these variants should be taken into consideration alongside genes such as *FTO*, *APOE*, or *TCF7L2*, as well as those involved in translocation (bilitranslocase’s gene, *SLC5A1*, *SLC2A2*, and *SLCO2B1*) and pre-absorption (*LCT* and *GBA3*) to ensure the optimal efficacy of the therapy.

### 6.3. The Microbial Mediator: The Gut Microbiome’s Central Role in Anthocyanin Metabolism and Efficacy

While genetics dictates the baseline capacity for gastrointestinal absorption, most ingested polyphenols (often over 95%) pass unabsorbed into the large intestine [195]. There, they encounter the gut microbiota, a dense and metabolically active community now recognized as a “metabolic organ” in its own right [196]. This microbial ecosystem transforms ACNs into a diverse array of secondary metabolites, which are often more bioactive and systemically available than their parent compounds [197]. Conversely, ACNs can modulate the composition and activity of the gut microbiota [198], highlighting a bidirectional relationship.

#### 6.3.1. Microbial Biotransformation: The Anthocyanin Bioactivation Engine

The gut microbiota harbors a vast enzymatic repertoire absent in human cells, enabling the catabolism of complex plant polyphenols, such as ACNs. Their microbial metabolism follows a series of well-defined steps, beginning with deglycosylation, the cleavage of the sugar moieties from the ACN glycosides [199]. This reaction, catalyzed by microbial β-glucosidases, β-glucuronidases, and α-L-rhamnosidases, which are prevalent in genera such as *Bifidobacterium* and *Lactobacillus* [200,201], releases the unstable aglycone form. Under the neutral-to-alkaline pH of the colon, the aglycone undergoes spontaneous C-ring fission, yielding simpler, more stable phenolic compounds: phloroglucinol derivatives from the A-ring and benzoic acid derivatives from the B-ring [202]. The resulting bioactive phenolic acids depend on the structure of the ACN. For example, cyanidin is primarily metabolized into PCA, vanillic, and p-coumaric acids [203]; malvidin produces syringic acid [204]; and delphinidin yields 2,4,6-trihydroxybenzaldehyde, gallic, and syringic acids [203].

This microbial transformation is not merely degradative but represents a bioactivation process. The resulting phenolic acids are smaller, less polar, and more readily absorbed from the colon into the bloodstream than their large, glycosylated precursors [66,205]. They often achieve higher and more sustained plasma and urine concentrations [206]. Crucially, these metabolites possess potent biological activity, often mediating positive effects, for example, at the cardiovascular level [207,208]. Evidence from animal models powerfully illustrates this dependency: black currant ACNs improved weight gain and glucose metabolism in mice with an intact gut microbiome but failed to do so in dysbiotic mice [209]. Thus, the health benefits of ACNs are intrinsically linked to the metabolic capacity and composition of the gut microbiota.

#### 6.3.2. The Prebiotic Effect: Anthocyanins as Modulators of the Gut Ecosystem

The interaction between ACNs and the gut microbiota is bidirectional. While microbiota metabolizes ACNs, these compounds and their metabolites also exert a selective pressure on the microbial community, acting as prebiotics [201,210]. ACN interventions lead to a significant proliferative effect on beneficial bacteria, particularly *Bifidobacterium* spp. [198] and *Lactobacillus* spp. [201], probiotics associated with improved gut barrier function and immune modulation [211], while inhibiting potentially pathogenic species, such as *Clostridium histolyticum* [198]. At a broader level, this prebiotic effect also leads to an alteration in community structure, exemplified by a reduction in the *Firmicutes*-to-*Bacteroidetes* (*F*/*B*) ratio. A high *F*/*B* ratio has been linked to obesity and metabolic dysbiosis [212]. This modulation also enhances production of short-chain fatty acids (SCFAs), primarily acetic acid, propionic acid, and butyric acid [212], which are highly beneficial for microbiome health [213].

MetS-associated diseases are often accompanied by gut imbalance or dysbiosis [214], characterized by a decrease in the abundance of beneficial, butyrate-producing bacteria and a concurrent increase in opportunistic pathogens [215]. This imbalance contributes to metabolic endotoxemia, as increased translocation of pro-inflammatory bacterial components like lipopolysaccharide impairs systemic metabolism [216]. In obesity, key species such as *Akkermansia muciniphila* and *Bifidobacterium animalis* are reduced [214], while T2DM is associated with lower levels of *Faecalibacterium*, *Roseburia*, and *Bifidobacterium* species [214]. ACNs counteract this disease-associated dysbiosis by selectively fostering the growth of beneficial bacteria (i.e., *Bifidobacterium* spp.), therefore restoring a healthier microbial community structure [198] and mitigating low-grade inflammation and metabolic dysfunction that underlie MetS.

Importantly, an individual’s baseline composition of microbiota strongly influences their response to ACN intervention. Individuals can be stratified based on their microbial profiles such as the *Prevotella*-to-*Bacteroides* (*P*/*B* ratio), which can predict health outcomes. For instance, a high *P*/*B* ratio correlates with greater improvements in glucose metabolism and fat loss, following high-fiber diets [217], suggesting that a *Prevotella*-dominant enterotype may represent a “responder” phenotype for certain interventions. The variability directly translates to differences in metabolic output, leading to the classification of individuals into “high” or “low” excretors of specific phenolic acid metabolites [218]. Individuals lacking the bacteria or enzymes required to produce key metabolites, such as PCA, may not gain the associated health benefits, thus being classified as “non-responders” from a functional perspective.

### 6.4. Influence of the Food Matrix and Processing on Anthocyanin Bioavailability

The biological fate of ACNs is influenced by the delivery system and the food matrix—the complex physical and chemical architecture of a food that shapes nutrient and bioavailability [219]. Food processing can alter this matrix, either enhancing or diminishing the potential health benefits of ACNs [83].

ACNs rarely occur in isolation; their interactions with macronutrients can either protect or inhibit them. Constituents can shield ACNs from the harsh pH conditions and enzymatic degradation in the digestive tract, increasing the amount reaching the colon for microbial metabolism. For instance, ACN levels from purple sweet potatoes and from red wine decreased by 27–43% and 49–52%, respectively, in the absence of matrix components, while glucose and proteins improved their stability [220]. This likely explains why whole-fruit matrix provides significantly higher bioavailability compared to a juice matrix [221]. However, excessive glucose or protein may impair intestinal transport [220]. Moreover, the chemical structure of the ACN also matters: acylated forms, common in vegetables like purple carrots and red cabbage, exhibit much lower plasma (8- to 10-fold) and urine (11- to 414-fold) recovery than non-acylated ones [222].

#### Impact of Food Processing

The journey from farm to table often involves processing steps that can fundamentally alter the food matrix and, consequently, ACN bioavailability. The effects of processing are not uniformly negative or positive but are highly context-dependent. Physical disruption such as juicing or puréeing can have mixed outcomes: juicing often lowers the bioavailability of ACNs by removing the protective fiber matrix [221], while minimal processing such as puréeing blanched blueberries was found to enhance the absorption of ACNs, possibly by breaking down cell walls and increasing their accessibility to digestive enzymes and transporters [223]. Thermal processing can also have variable effects: cooking purple carrots increased urinary recovery of non-acylated ACNs but not of the poorly absorbed acylated forms [222]. Similarly, fermentation can reduce the bioavailability of the parent ACN by over 10% in red cabbage compared to its fresh counterpart [224]. In contrast, modern food technology like encapsulation and fortification can enhance bioavailability. Encapsulation carriers such as whey protein or citrus pectin, or nanoencapsulation, can shield ACNs from degradation and allow controlled release in the gastrointestinal tract, and improve absorption kinetics [206]. In this context, plant-derived exosome-like nanovesicles (PDENs) emerge as a novel natural nanoencapsulation system [225]. These carriers enhance ACN stability and bioavailability, with demonstrated hepatoprotective effects: grape GELNs inhibit NLRP3/TNF-α pathways in liver injury models, while blueberry BELNs reduce NAFLD lipogenesis and inflammation (IL-6/TNF-α) [226]. Within precision nutrition frameworks, PDENs offer patient-specific ACN delivery, bridging food matrix optimization with targeted metabolic interventions.

Overall, the superior nutritional value of whole foods over processed forms or isolated extracts is particularly evident for ACNs. Whole fruits provide ACNs within a fibrous matrix that not only protects them but also supports the gut microbiome essential for their metabolism; this synergy is lost in juices or extracts. Thus, within a precision nutrition framework, the food source and degree of processing are key determinants of the effective dose and bioactive potential of ACNs.

### 6.5. Leveraging Metabolomics to Determine Metabolic Signature for True Personalization

While genomics reveals an individual’s potential to transport and metabolize ACNs and the metagenome reflects the functional capacity of their microbiome, metabolomics provides a real-time, functional snapshot of physiological function. By profiling metabolites in biological fluids such as plasma or urine, metabolomics captures the integrated output of the interactions between genes, diet, microbiome, and lifestyle.

#### Metabolomics for Objective Biomarker Discovery

A major challenge in nutritional science is the reliance on self-reported dietary intake, which is prone to error and bias. Metabolomics offers a solution by enabling the discovery of objective biomarkers that reflect actual consumption and metabolic response [227]. For example, untargeted metabolomic analysis through ultra-performance liquid chromatography–mass spectrometry (UPLC-MS) of plasma has identified salsolinol sulfate and 4-methylcatechol sulfate as biomarkers of ACN-rich berry intake; these biomarkers were also inversely correlated with visceral adipose tissue, indicating improved cardiometabolic health [228]. Similarly, 2-furoylglycine, alkylresorcinols, and resveratrol metabolites (in urine) have been validated as biomarkers for coffee [229], whole-grain wheat [230], and wine [231], respectively. Such biomarkers provide objective measures of exposure that account for bioavailability, a major advantage over dietary questionnaires. Beyond confirming intake, metabolomics can uncover biomarkers of effect, revealing how ACN interventions modulate endogenous metabolism. A metabolomics study through ^1^H-NMR showed that ACN consumption can alter metabolites linked to the tricarboxylic acid (TCA) cycle, gut microbial metabolism, and renal function [232], providing mechanistic insights into their physiological benefits.

The recognition that individuals respond differently to the same diet has led to the concept of “metabotyping”—stratifying individuals into groups based on their metabolic profiles [233]. These metabotypes can predict responsiveness to nutritional intervention, allowing the identification of “responders” and “non-responders” [233]. A notable example comes from a randomized controlled trial on Korean black raspberry supplementation, where baseline urinary metabolomics predicted antioxidant response. Higher baseline levels of glycine and N-phenylacetylglycine were significantly correlated with a greater improvement in the GSH/glutathione disulfide (GSSG) ratio, a key marker of antioxidant status, after the four weeks [234]. Such findings demonstrate the predictive value of baseline metabolic profiles. Integrating a patient’s genotype, enterotype, and metabotype could ultimately enable precise, personalized dietary recommendations, marking a significant advance beyond one-size-fits-all nutrition strategies.

### 6.6. Integrating Lifestyle Factors into the Precision Nutrition Framework to Achieve a Holistic View

A comprehensive precision nutrition framework must acknowledge that dietary interventions do not occur in a physiological vacuum. An individual’s response to ACNs is not determined by genetics or microbiome composition alone but rather by an integrated phenotype—the combined and interactive effects of genetic, microbial, metabolic, and lifestyle and environmental factors [235]. Neglecting this broader context can produce inconsistent findings in clinical trials and limit the effectiveness of personalized dietary strategies. For example, two individuals may share the same “responder” genotypes for key ACN transporters yet exhibit markedly different outcomes depending on their lifestyle. A physically active person tends to display a healthier gut microbial profile, including a better *P*/*B* ratio [236], which enhances responsiveness to ACNs. In contrast, individuals with overweight, obesity, or diabetes frequently present a dysbiotic gut microbiota [214,237], which may actively counteract or negate ACN-mediated benefits. This interaction explains much of the variability observed even under tightly controlled nutritional studies and underscores the need to assess the individual as a dynamic system rather than in isolation. Similarly, the response to a nutritional intervention can vary depending on when it is consumed relative to an individual’s internal clock and external cues like the light–dark cycle—a concept known as chrononutrition [238]. Misalignment between eating patterns and circadian rhythms, common in shift workers or individuals with irregular eating habits, is associated with an increased risk of metabolic disorders [239]. Therefore, the timing of ACN consumption may be a critical variable that interacts with an individual’s chronotype to influence metabolic outcomes.

### 6.7. Towards an Integrated Model of Precision Nutrition

The profound interindividual variability in response to dietary ACNs is not a barrier for precision nutrition but rather an opportunity to adopt a more sophisticated, tailored approach. An individual’s response emerges as a predictable, multifactorial outcome shaped by at least five key domains: the genetic blueprint, which sets the inherent ADME capacity; the microbial mediator, which acts as a crucial bioactivation engine; the food matrix, which controls dose; and the metabolic signature, which provides a real-time functional readout of the system; and the lifestyle context, which modulates overall responsiveness.

A “responder” is likely someone with a favorable combination of these factors: a genetic profile that enables efficient absorption or colonic delivery, microbiome that converts ACNs into highly bioactive phenolic acids, a diet that delivers these compounds in a protective and synergistic whole-food matrix, a receptive metabolic baseline, and a lifestyle that promotes metabolic health.

Future progress will depend on moving away from single-factor analyses and toward integrated, multi-omics models that capture data from each domain simultaneously. Predictive algorithms that weigh genetic variants, microbial enterotype, metabotype, and lifestyle will be crucial for designing truly personalized and effective nutritional strategies.

**Figure 3:**
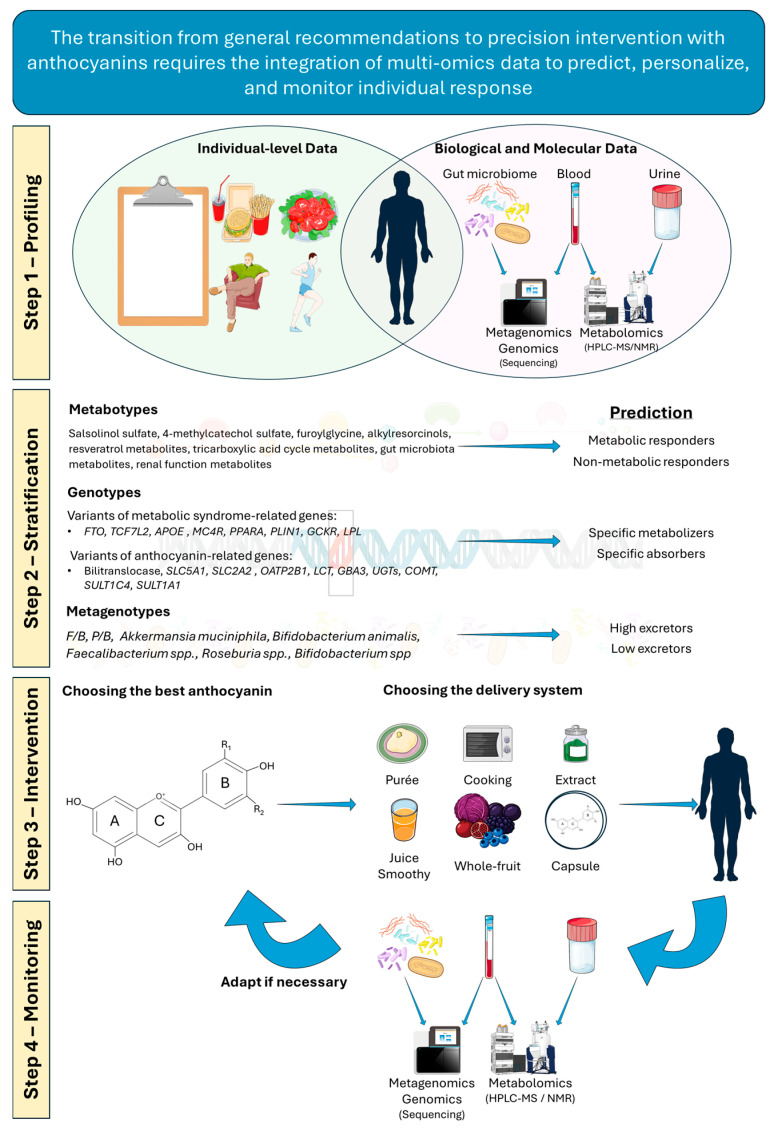
A multi-omics framework for precision anthocyanin intervention. This workflow outlines the integration of individual-level phenotypic data (lifestyle, diet, anthropometry) with deep molecular profiling obtained through metagenomics, genomics, and metabolomics approaches. Based on this data, individuals are classified to predict therapeutic outcomes, relying on metabotypes, genotypes, and metagenotypes to distinguish responders from non-responders. This would allow the design of a tailored regimen involving the selection of the specific anthocyanin structure and the most effective delivery system. Application relies on a continuous feedback loop where post-intervention multi-omics data is used to assess efficacy and adapt the strategy if necessary. Abbreviations: *F*/*B: Firmicutes*-to-*Bacteroidetes* ratio; HPLC-MS: high-performance liquid chromatography coupled with mass spectrometry; NMR: nuclear magnetic resonance; *P*/*B*: *Prevotella*-to-*Bacteroides* ratio. Adapted from Servier Medical Art (https://smart.servier.com), licensed under CC BY 4.0 (https://creativecommons.org/licenses/by/4.0/).

Looking forward, integrating multi-omics data with in silico approaches holds great promise for identifying specific molecular targets of ACNs. Combining patient-specific metabolomics (phenolic acid profiles) with molecular dynamics simulations could predict individual target engagement, transforming pleiotropic nutraceuticals into precision therapeutics. By embracing this complexity, precision nutrition can deliver the right nutrient, in the right form, at the right time, to the right person, optimizing metabolic health (Figure 3).

## 7. Clinical Evidence and Therapeutic Potential in Metabolic Medicine

Table 1 and Table 2 compile pre-clinical and clinical studies, respectively, assessing the effects of ACN-rich interventions. This comprehensive body of evidence establishes the physiological benefits of ACN consumption, demonstrating multi-level convergence, where functional benefits observed in human subjects are consistently validated by underlying molecular and cellular changes identified in animal models.

### 7.1. Model and Population Selection

These studies employ various methodologies, defining key differences in intervention delivery, dose, and duration, which are essential for accurately mapping the full spectrum of ACN bioactivity, from rapid vascular relaxation to long-term modulation of the gut–liver axis. In terms of model and population, clinical trials focused on established risk factors and chronic conditions, such as hypercholesterolemia [245,246], MetS [247,248,249,251], obesity [250,252], and IR [253], whereas pre-clinical models mirrored these conditions for mechanistic validation. For instance, the use of Wistar rats [240] and C57BL/6J mice [241,242,243] fed an HFD, or New Zealand rabbits [244] on a 1% cholesterol diet, provided pathological contexts for the above-mentioned conditions. Another key design element was the use of established therapeutic agents as positive controls—atorvastatin [240], simvastatin [244], and metformin [243]—which allowed for the establishment of robust benchmarks for the efficacy of ACN interventions.

### 7.2. Intervention Delivery, Duration, and Dosage

Regarding delivery, dose, and duration, these were chosen based on the desired endpoint. Studies utilized diverse delivery systems, from whole foods/juices (e.g., 80 g daily, red-fleshed apples (RFAs) [245], 600 g/day whole blackberries [250] to high-concentration processed forms (e.g., aronia extract [245], cornelian cherry powder/extract [244,253]), highlighting the matrix effect. This refers to how the whole food and all of its constituents influence the effect of a bioactive compound. The apple-based interventions [240,245,246] demonstrated that the aronia liquid matrix allowed for better absorption of ACNs. Conversely, the whole apples may inhibit absorption, suggesting that pectin fiber may limit the uptake of ACNs, limiting systemic bioavailability. Despite this potential inhibition, the whole-food matrix provided unique benefits, underscoring the contribution of non-ACN compounds. Specifically, one study noted that RFA showed better anti-inflammatory effects than aronia, despite a similar content of ACNs [245]; furthermore, whole, white-fleshed apples (WFAs) with minimal ACN content still provided significant cardiovascular results [240,245,246]. This possibly suggests that other polyphenols or fiber may provide a protective synergistic effect. Duration and dose also varied: acute or short-term designs [249,252] were used to assess rapid effects, such as the strawberry beverage reducing postprandial insulin area under the curve (AUC) (0–6 h) without affecting glucose [252], while chronic/systemic designs were used to measure structural improvements like dose-specific effects: 1 cup/day of blueberries produced clinically meaningful cardiometabolic benefits, while ½ cup/day was insufficient and even raised TG levels [248].

### 7.3. Multi-Level Convergence of Physiological Results

#### 7.3.1. Immunomodulatory and Antioxidant Effects

ACN interventions exert potential anti-inflammatory effects by modulating systemic immune pathways, the biological basis for all observed health benefits. Study results consistently showed the suppression of key inflammatory markers: RFA and aronia downregulated CRP and IL-6 [245], while the strawberry beverage attenuated postprandial IL-6 and CRP elevations [252]. Pre-clinical evidence showed cytokine suppression by lowering levels of IL-6, TNF-α, IL-1 β, and NF-κB gene expression after blueberry intervention [242,243]. It is also noteworthy that WFA led to the downregulation of iron-binding proteins, contributing to reduced OS and preserved NO bioavailability [240].

#### 7.3.2. Cardiovascular Health and Vascular Protection

Results demonstrated a multi-level convergence. For instance, ACN intervention consistently improved vascular function and reduced key inflammatory drivers, with pre-clinical results supporting efficacy comparable to statins. Clinical findings showed RFA improved arterial vasodilation (suggesting enhanced NO production) [245], and blueberry consumption improved endothelial function (↑ flow-mediated dilation (FMD), ↑ reactive hyperemia index (RHI)) and reduced arterial stiffness (↓ augmentation index (AIx)) [248].

This efficacy is validated by molecular and structural evidence from pre-clinical models, using two distinct statin benchmarks. Cherry extract reduced the intima/media ratio in the aorta of rabbits, a similar effect to that of simvastatin, confirming the potential vascular protective role of ACNs [244]. Furthermore, the apple interventions, one of which included atorvastatin, helped confirm the anti-atherosclerotic effect of WFA. These promoted the downregulation of complement system proteins (C1q, C4, Factor B, and C3) and evidenced stronger complement suppression compared to RFA [240,245].

#### 7.3.3. Metabolic Regulation and Glycemic Control

ACNs demonstrated powerful multi-target effects on glucose–insulin dynamics, with pre-clinical results supporting superior efficacy to metformin. Clinical data confirmed that chronic ACN intake, particularly cherry and strawberry, improves IS by reducing fasting insulin and Homeostasis Model Assessment—Insulin Resistance (HOMA-IR) [251,253]. Specifically, cherry powder in conjunction with Medical Nutrition Therapy (MNT) produced the greatest improvements in fasting glucose, insulin, C-peptide, and HOMA-IR, achieving an average HOMA-IR below the diagnostic cut-off [253]. Moreover, blueberry and strawberry successfully blunted postprandial dysregulation and reduced postprandial insulin AUC following high-fat/high-sugar meals [252].

These clinical findings are also validated by pre-clinical evidence, specifically through the metformin benchmark: ACNs from blackberries demonstrated superior performance in improving oral glucose and insulin tolerance tests in mice, alongside significant reduction in glucose, insulin, HOMA-IR, and glycated hemoglobin (HbA1c) [243]. Notably, ACNs from blackberries improved insulin signaling pathway and GSH metabolism [242], structurally supporting the clinical reduction in IR.

#### 7.3.4. Lipid Metabolism, Body Composition, and the Gut–Liver Axis

These bioactive compounds modulated lipid profiles across species and improved metabolic efficiency in humans, largely driven by the gut–liver axis. Trial outcomes showed chronic lipid benefits, most notably with blueberry consumption leading to a significant increase in HDL-cholesterol, APOA-I, and HDL particle number [248], whereas whole apples lowered total cholesterol (TC), LDL-cholesterol, and TG [246].

Animal model data from blackberry and blueberry interventions demonstrated that ACNs prevented HFD-induced weight gain and induced favorable shifts in fat depots—lowering white adipose tissue while increasing brown adipose tissue [243]. It is worth noting that serum and hepatic TC, TG, and LDL-cholesterol were reduced, and HDL-cholesterol was enhanced [241,242].

#### 7.3.5. Specialized Hepatic and Endocrine Mechanisms

Beyond core metabolic outcomes, ACNs demonstrated critical modulatory roles in specialized physiological systems. A high-dose strawberry intake revealed a decrease in branched-chain amino acids (e.g., valine and leucine [251]) strongly linked to metabolic dysfunction and IR. Changes in mitochondrial activity were also observed, mainly an increase in serum phosphate and glycerol-phosphate, markers of enhanced energy generation and mitochondrial activity. Pre-clinical data using cornelian cherry extract elucidated a key endocrine and transcriptional mechanism for cardiometabolic protection; the extract led to an increase in the mRNA expression of PPARα and PPARγ in the aorta, as well as liver X receptor α (LXRα) in the liver. This treatment also caused a favorable shift in adipokines, i.e., a decrease in leptin and resistin (pro-inflammatory) and an increase in adiponectin (anti-inflammatory) [244]. This directly reinforces the anti-inflammatory and insulin-sensitizing effects, supporting the observed clinical reductions in TC and HOMA-IR.

Overall, the body of clinical and pre-clinical evidence consistently establishes a therapeutic potential of ACN-rich interventions in metabolic medicine. The multi-level convergence of results, from the suppression of inflammatory markers and enhanced vascular function to improvements in glycemic control and lipid profiles, underlines an integrated mechanism of action. Notably, the observed benefits are comparable or even superior to standard-of-care agents such as statins and metformin. Moreover, the findings on the matrix effect further underscore the complex nature of food-based therapeutics, such as precision nutrition. This extensive validation confirms that ACNs and their food matrices represent a highly promising, multi-target framework for the prevention and management of MetS and related pathologies.

## 8. Conclusions

The increase in MetS, caused by chronic inflammation and OS, makes personalized dietary strategies essential. ACNs are a promising nutritional tool due to their pleiotropic effect, which improves insulin sensitivity, optimizes lipid metabolism, and supports cardiovascular function. Clinical efficacy is limited by their low bioavailability and interindividual variability in response, influenced by the interaction between gut microbiota and genetic polymorphisms. Therefore, to fully exploit the potential of ACNs, it is necessary to shift to a precision nutrition paradigm that, through the integration of multi-omics data, such as genomics, metagenomics, and metabolomics, can personalize intervention and maximize individual metabolic benefits.

## Figures and Tables

**Table 1:** Pre-clinical evidence on the efficacy of anthocyanin-rich fruits on metabolic syndrome and associated pathologies.

Target Disease	Study Design	Main Findings
**Apple ACNs**		
Hypercholesterolemia	**Pre-clinical:** **Animal model:** Wistar rats (*n* = 36 (3 females, 3 males/group). **Duration:** 9 wks (3 wks HFD induction + 6 wks intervention). **Intervention details**: SCD, HFD, HFD + RFA, HFD + WFA, HFD + aronia extract, HFD + atorvastatin (human equivalent of 70 mg/day ACN content) [240].	**Inflammation and OS:** ↓ complement system proteins [240]. **Vascular Findings:** direct changes in aortic tissue proteins [240]. **Matrix Effects:** RFA and WFA showed cardiovascular benefits despite different ACN content. RFA showed better anti-inflammatory effects than aronia. Whole apples with minimal ACNs still provided significant cardiovascular benefits [240].
**Blueberry ACNs**		
MetS	**Pre-clinical:** **Animal model:** C57BL/6J mice (*n* = 27 (13 females and 14 males, 8/group). **Duration:** 8 wks. **Intervention details:** SCD (oral gavage of saline) vs. HFD (oral gavage of saline) vs. HFD + blueberry ACN extract (oral gavage of 100 mg/kg every other day) [241]	**Vascular Function:** Not directly assessed (liver/gut focus); vascular benefit inferred from ↑ metabolic/liver profile [241]. **Lipid Metabolism:** ↓ serum cholesterol, ↓ hepatic TG, lipid-lowering in HFD [241]. **Glucose and Insulin Homeostasis**: ↓ fasting glucose; ↓ fasting insulin; ↓ HOMA-IR [241]. **Liver Function:** hepatoprotection (↓ ALT/AST, steatosis, ↑ liver histology) [241] **Inflammation & OS:** ↓ hepatic TNF-α, ↓ IL-6, ↓ OS markers [241]. **Gut Microbiota and Bile Acids:** clear microbiota shifts (↑ Akkermansia, ↓ obesogenic taxa); bile acid pools altered (↑ beneficial bile acids activating FXR/TGR5) [241].
**Blackberry ACNs**		
Obesity	**Pre-clinical:** **Animal model:** male C57BL/6J mice (*n* = 60 (12/group)). **Duration:** 12 wks. **Intervention details:** LFD vs. HFD vs. HFD + orlistat vs. HFD + Blackberry ACN vs. HFD + Blueberry ACN (ACN content results in human equivalent of ~2 mg/kg of body weight) [242]. **Pre-clinical:** **Animal model:** male C57BL/6J mice (*n* =32, 8 SCD, 24 HFD). **Duration:** 17 wks total (1 wk acclimation, 8 wks HFD induction, 8 wks intervention). **Intervention details:** SCD or HFD for 8 wks, after that, mice divided in 3 groups: HFD (gavaged with water) vs. HFD + ACN (gavaged with 100 mg/kg mixture of blueberry and blackberry acns vs. HFD + Metformin (gavaged with 100 mg/kg metformin) [243].	**Obesity and Weight Control:** both fruits prevented HFD-induced weight gain [242,243]; shift in fat depots (↓ WAT, ↑ BAT) [243]. **Lipid Metabolism:** ↓ TC, TG, LDL-cholesterol, ↑ HDL-cholesterol [242,243]. **Glucose and Insulin:** ↑ insulin signaling [242]; ↓ glucose, insulin, HOMA-IR, HbA1c, ↑ tolerance tests (even > metformin) [243]. **Inflammation & OS:** ↓ TNF-α, IL-6/IL-1β, NF-κB; ↑ SOD, GPx; ↓ MDA [242,243]. **Gut Microbiota and Metabolites:** ↑ SCFAs (acetate, propionate, butyrate) [242]; selective ↑ acetic acid, ↑ *Prevotella histicola*; FMT confirmed causality [243]. **Energy Metabolism:** Hepatic metabolomics → ↑ glycerophospholipid, GSH, insulin signaling [242], ↑ BAT mass, ↑ gut–liver metabolic cross-talk [243].
**Cherry ACNs**		
Dyslipidemia	**Pre-clinical:** **Animal model:** male New Zealand rabbits (*n* = 50 (10/group)). **Duration:** 4 wk adaptation period, 60 intervention days. **Intervention details:** SCD vs. SCD + 1% cholesterol vs. SCD + 1% cholesterol + cherry extract (10 mg/kg of bodyweight) vs. SCD + 1% cholesterol + cherry extract (50 mg/kg of bodyweight) vs. SCD + 1% cholesterol + simvastatin (5 mg/kg of bodyweight) [244].	**Metabolic Health and IS:** Extract supplementation ↓ HOMA-IR, glucose and insulin; ↑ adiponectin, ↓ leptin and resistin [244]. **Lipid and Adipokine Modulation:** Cherry ↓TG and attenuated cholesterol-induced hyperlipidemia and ↑ PPARα and PPARγ in the aorta and LXRα in the liver [244]. **Vascular Protection:** Cherry ↓ the aortic intima/media ratio- ↓ vascular wall thickening and early atherosclerosis; effects comparable to simvastatin in magnitude [244].

Abbreviations: ACN, anthocyanin; *n*, sample size; wk, week; HFD, high-fat diet; SCD, Standard Chow Diet; RFA, red-fleshed apples; WFA, white-fleshed apples; OS, oxidative stress; MetS, metabolic syndrome; TG, triglycerides; HOMA—IR, Homeostasis Model Assessment of Insulin Resistance; ALT/AST, Alanine Aminotransferase/Aspartate Aminotransferase; TNF—α, Tumor Necrosis Factor alpha; IL-6, Interleukin-6; FXR/TGR5 Farnesoid X Receptor/Takeda G-protein-coupled Receptor 5; WAT, white adipose tissue; BAT, brown adipose tissue; TC, total cholesterol; LDL, low-density lipoprotein; HDL, high-density lipoprotein; HbA1c, hemoglobin A1c; IL-1β, Interleukin-1 beta; NF—κB, Nuclear Factor κB; SOD, superoxide dismutase; GPx, glutathione peroxidase; MDA, malondialdehyde; SCFAs, short-chain fatty acids; FMT, fecal microbiota transplantation; GSH, Glutathione; PPARα/PPARγ, peroxisome proliferator-activated receptor alpha/gamma; LXRα, liver X receptor alpha; ↓, downregulation/decrease; ↑, upregulation/increase.

**Table 2:** Clinical evidence on the efficacy of anthocyanin-rich fruits on metabolic syndrome and associated pathologies.

Target Disease	Study Design	Main Findings
**Apple ACNs**		
Hypercholesterolemia	**RCT:** **Population characteristics:** adults with LDL-cholesterol ≥ 115 mg/dL (*n* = 121, 41 WFA (68% female, age: 49.8 ± 13.6 y, BMI: 24.6 ± 3.2 kg/m^2^), 40 aronia infusion (50% female, age: 49.6 ± 13.3 y, BMI: 26.3 ± 4.5 kg/m^2^), RFA (54% female, age: 46.7 ± 16.3 y, BMI: 26.3 ± 3.8 kg/m^2^); **Duration:** 6 wks. **Intervention details:** daily RFA (34.5 mg ACNs) vs. daily WFA (0 mg ACNs) vs. daily aronia infusion (37.4 mg ACNs) [245].	**Inflammation & OS:** in blood: ↓ CRP, IL-6, ICAM-1 [245,246]. **Vascular Findings:** ↑ endothelial function (measured by ischemic reactive hyperemia); ↑ microvascular vasodilation; ↓ adhesion molecules [245,246]. **Matrix Effects:** RFA and WFA showed cardiovascular benefits despite different ACN content. RFA showed better anti-inflammatory effects than aronia. Whole apples with minimal ACNs still provided significant cardiovascular benefits [245,246].
	**RCT (crossover):** **Population Characteristics:** healthy adults with mild hypercholesterolemia (*n* = 40, 58% female, age: 51 ± 11 y, BMI: 25.3 ± 3.7 kg/m^2^). **Duration:** 20 wks total (8 wks per intervention, 4 wks washout period). **Intervention details:** whole apples (ACNs as 1–3% of total polyphenols) vs. sugar-matched control beverage [246].	
**Blueberry ACNs**		
MetS	**RCT:** **Population characteristics:** adults with MetS (*n* = 44, 23 blueberries (52% female, age: 55 ± 2 y, BMI 35.2 ± 0.8 kg/m^2^), 21 placebo (76% female, age: 59 ± 2 y, BMI: 36.0 ± 1.1 kg/m^2^)). **Duration:** 6 wks. **Intervention details:** twice daily smoothie (290.3 g ACNs) vs. placebo smoothie (0 mg ACNs) [247].	**Vascular Function:**↑ endothelial function (↑ FMD, RHI); ↑ vascular signaling (↑ cGMP); ↓ arterial stiffness (↓ AIx); no consistent BP changes [247,248]. **Lipid Metabolism:** ↑ HDL, ↑ ApoA-I, ↑ HDL particles [248]; HDL-cholesterol + ApoA-I preserved postprandially [249]. **Glucose and Insulin Homeostasis**: no improvements chronically [247,248]; blunted glucose/insulin spikes post-meal [249].
	**RCT:** **Population characteristics:** adults with MetS (*n* = 45, 34% female, age: 63.4 ± 7.4 y, BMI: 31.4 ± 3.1 kg/m^2^). **Duration:** 24 h (acute, postprandial). **Intervention details:** energy-dense drink with blueberry powder (364 mg ACN) vs. placebo powder (0 mg ACNs) [249].	
	**RCT:** **Population characteristics:** overweight/obese (BMI ≥ 25 kg/m^2^) adults with MetS (*n* = 115, 32% female, age: 63 ± 7 y, BMI: 31.2 ± 3.0 kg/m^2^). **Duration:** 6 months. **Intervention details:** daily consumption of 1 cup (364 mg ACN) vs. 1/2 cup (182 mg ACN) vs. placebo (0 mg ACN) [248].	
**Blackberry ACNs**		
Obesity	**RCT (crossover):** **Population Characteristics:** Overweight/obese men (BMI > 25 kg/m^2^) (*n* = 27, 24 included in glucose metabolism analysis (age: 57.8 ± 2.1 y, BMI: 30.6 ± 0.8 kg/m^2^), 17 in calorimetry analysis (age: 61 ± 1.9 y, BMI: 30.7 ± 1 kg/m^2^). **Duration:** 3 wks total (1 wk per intervention, 2 interventions, 1 wk washout period). **Intervention details:** HFD + whole blackberries (~361 mg ACNs) vs. HFD + calorically matched gelatin [250].	**Obesity and Weight Control:** no weight loss in 7 days, but ↑ fat oxidation suggests potential long-term weight control effect [250]. **Glucose and Insulin:** no glucose effect, ↓ insulin AUC, ↓ HOMA-IR [250]. **Inflammation and OS:** not measured, but reduced insulin suggests improved metabolic inflammation [250]. **Energy Metabolism:** ↑ fat oxidation (↓ RQ) [250].
**Strawberry ACNs**		
MetS &Obesity	**RCT (crossover):** **Population characteristics:** adults with MetS (*n* = 33, 94% female, age 53 ± 13 y, BMI 33 ± 3 kg/m). **Duration:** 14 wks (4 wks per intervention, 3 interventions, 1 wk washout period). **Intervention details:** Control powder (calorically equivalent, 0 mg ACNs) vs. high-dose strawberry (~92 mg ACNs) vs. low-dose ACNs (~38 mg ACNs) [251].	**Insulin Regulation:** Both interventions ↑ IS [251,252]; either by ↓ postprandial insulin peaks [252], or ↓ fasting insulin/HOMA-IR [251]. **Anti-inflammatory and Antioxidant Effects:** Both show suppression of inflammatory stress; either by immediate ↓ cytokine [252] or by showing metabolomic signatures consistent with ↓ inflammation [251]. **ACN-Driven Mechanisms:** Pelargonidin-based ACNs act as primary bioactives—evidence of microbial and systemic metabolism in both studies. **Cardiometabolic Target:** Both improve early and established markers of metabolic dysfunction (postprandial stress and MetS).
	**RCT (crossover):** **Population Characteristics:** Overweight/obese adults (*n* = 24, 59% female, age: 50.9 ± 15.0 y, BMI: 29.2 ± 2.3 kg/m^2^). **Duration:** 6 h postprandial trial, 2 test days, 3–5 days apart. **Intervention details:** high carbohydrate, moderate fat meal + strawberry beverage 81.6 ± 5.6 mg ACNs) vs. high carbohydrate, moderate fat meal + placebo beverage (0 mg ACNs) [252].	
**Cherry ACNs**		
IR	**RCT:** **Population characteristics:** women with IR (*n* = 84, median age: 35 y). **Duration:** 12 wks. **Intervention details:** MNT vs. Cherry powder (237.55 mg ACNs) vs. MNT + Cherry powder vs. Control [253]	**Metabolic Health and IS:** Cherry intake ↓ fasting glucose, insulin, C-peptide, HOMA-IR; MNT + cherry yielded ~2× greater ↑ than diet therapy alone and normalized HOMA-IR (<2.5) [253]. **Lipid and Adipokine Modulation:** Cherry intake ↑ metabolic profile, ↓ in body weight, waist circumference [253].

Abbreviations: ACN, anthocyanin; RCT, randomized controlled trial; *n*, sample size; wk, week; HFD, high-fat diet; SCD, Standard Chow Diet; RFA, red-fleshed apples; WFA, white-fleshed apples; CRP, C-reactive protein; IL-6, Interleukin-6; ICAM-1, Intercellular Adhesion Molecule 1; OS, oxidative stress; LDL, low-density lipoprotein; BMI, body mass index; MetS, metabolic syndrome; FMD, flow-mediated dilation; RHI, reactive hyperemia index; cGMP, Cyclic Guanosine Monophosphate; AIx, augmentation index; BP, blood pressure; TG, triglycerides; HDL, high-density lipoprotein; ApoA-I, apolipoprotein A—I; HOMA—IR, Homeostasis Model Assessment of Insulin Resistance; IS, insulin sensitivity; ALT/AST, Alanine Aminotransferase/Aspartate Aminotransferase; TNF—α, Tumor Necrosis Factor α; NO, nitric oxide; FXR/TGR5 Farnesoid X Receptor/Takeda G-protein-coupled Receptor 5; LFD, low-fat diet; WAT, white adipose tissue; BAT, brown adipose tissue; TC, total cholesterol; HbA1c, hemoglobin A1c; AUC, area under the curve; IL-1β, Interleukin-1β-, NF—κB, Nuclear Factor κB; SOD, superoxide dismutase; GPx, glutathione peroxidase; MDA, malondialdehyde; SCFAs, short-chain fatty acids; IR, insulin resistance; MNT, Medical Nutrition Therapy; PPARα/PPARγ, peroxisome proliferator-activated receptor α/γ; LXRα, liver X receptor α; ↓, downregulation/decrease; ↑, upregulation/increase.

## Data Availability

No new data were created or analyzed in this study. Data sharing is not applicable to this article.

## Abbreviations

The following abbreviations are used in this manuscript:

AACE	Association of Clinical Endocrinologists
ACN	Anthocyanin
ADA	American Diabetes Association
ADME	Absorption, Distribution, Metabolism, and Excretion
AGEs	Advanced Glycation End Products
AHA	American Heart Association
AIx	Augmentation Index
AMPK	AMP-Activated Protein Kinase
AP-1	Activator Protein 1
APO	Apolipoprotein
AUC	Area Under the Curve
BMI	Body Mass Index
C/EBPα	CCAAT/Enhancer-Binding Protein α
C3G	Cyanidin-3-O-Glucoside
CAT	Catalase
COMT	Catechol-O-methyltransferase
COX2	Cyclooxygenase-2
DASH	Dietary Approach to Stop Hypertension
DGA	Dietary Guidelines for Americans
eNOS	Endothelial Nitric Oxide Synthase
ERK	Extracellular signal-Regulated Kinase
FMD	Flow-Mediated Dilation
GLUT	Glucose Transporter
GPx	Glutathione Peroxidase
GSH	Glutathione
GSSG	Glutathione Disulfide
HAT	Hydrogen atom transfer
HbA1c	Hemoglobin A1c
HDL	High-Density Lipoprotein
HFD	High-Fat Diet
HOMA	Homeostasis Model Assessment—Insulin Resistance
ICAM-1	Intercellular Cell Adhesion Molecule
IFN-γ	Interferon γ
IKK	IκB kinase
IL	Interleukin
iNOS	Inducible Nitric Oxide Synthase
IR	Insulin Resistance
IRS-1	Insulin Receptor Substrate-1
IS	Insulin Sensitivity
LDL	Low-Density Lipoprotein
LPH	Lactase-Phlorizin Hydrolase
LXRα	Liver X Receptor α
MAPK	Mitogen-Activated Protein Kinase
MetS	Metabolic Syndrome
MNT	Medical Nutrition Therapy
NF-κB	Nuclear Factor-κB
NHLBI	National Heart, Lung, and Blood Institute
NO	Nitric Oxide
Nrf2	Nuclear Factor Erythroid 2-Related Factor 2
O^2•−^	Superoxide
OATP2B1	Organic Anion Transporting Polypeptide 2B1
OS	Oxidative Stress
PCA	Protocatechuic Acid
PI3K	Phosphoinositide 3-Kinase
PKB or Akt	Protein kinase B
PPARγ	Peroxisome Proliferator-Activated Receptor γ
RFA	Red-Fleshed Apples
RHI	Reactive Hyperemia Index
RNS	Reactive Nitrogen Species
ROS	Reactive Oxygen Species
SCD	Standard Chow Diet
SCFAs	Short-Chain Fatty Acids
SET	Single-Electron Transfer
SFAs	Saturated Fatty Acids
SGLT1	Sodium–Glucose Cotransporter 1
SNPs	Single-Nucleotide Polymorphisms
SOD	Superoxide Dismutase
SREBP-1c	Sterol Regulatory Element-Binding Protein-1c
SULTs	Sulfotransferases
T2DM	Type 2 Diabetes Mellitus
TC	Total Cholesterol
TG	Triglycerides
TMA	Trimethylamine
TMAO	Trimethylamine N-Oxide
TNF-α	Tumor Necrosis Factor α
UGTs	UDP-Glycosyltransferases
UPLC-MS	Ultra-Performance Liquid Chromatography–Mass Spectrometry
VCAM-1	Vascular Cell Adhesion Molecule
WFA	White-Fleshed Apples
WHO	World Health Organization
WT	Wild-Type
